# Combination of mTORC1/2 inhibitor vistusertib plus fulvestrant in vitro and in vivo targets oestrogen receptor-positive endocrine-resistant breast cancer

**DOI:** 10.1186/s13058-019-1222-0

**Published:** 2019-12-04

**Authors:** Sunil Pancholi, Mariana Ferreira Leal, Ricardo Ribas, Nikiana Simigdala, Eugene Schuster, Sophie Chateau-Joubert, Lila Zabaglo, Margaret Hills, Andrew Dodson, Qiong Gao, Stephen R. Johnston, Mitch Dowsett, Sabina C. Cosulich, Elisabetta Maragoni, Lesley-Ann Martin

**Affiliations:** 1 Breast Cancer Now Toby Robins Research Centre, The Institute of Cancer Research, London, SW7 3RP UK; 2 BioPole Alfort, Ecole Nationale Veterinaire d’Alford, Maisons Alfort, France; 3 Ralph Lauren Centre for Breast Cancer Research, Royal Marsden Hospital, London, SW3 6JJ UK; 4 Breast Unit, Royal Marsden Hospital, London, SW3 6JJ UK; 5 Bioscience, Oncology, IMED Biotech Unit, AstraZeneca, Cambridge, UK; 6 Department of Translational Research, Institut Curie, Paris, France

**Keywords:** Breast cancer, Oestrogen receptor, mTORC1/2 signalling, Vistusertib, Endocrine resistance

## Abstract

**Background:**

Endocrine therapies are still the main strategy for the treatment of oestrogen receptor-positive (ER+) breast cancers (BC), but resistance remains problematic. Cross-talk between ER and PI3K/AKT/mTORC has been associated with ligand-independent transcription of ER. We have previously reported the anti-proliferative effects of the combination of everolimus (an mTORC1 inhibitor) with endocrine therapy in resistance models, but potential routes of escape via AKT signalling can lead to resistance; therefore, the use of dual mTORC1/2 inhibitors has met with significant interest.

**Methods:**

To address this, we tested the effect of vistusertib, a dual mTORC1 and mTORC2 inhibitor, in a panel of endocrine-resistant and endocrine-sensitive ER+ BC cell lines, with varying *PTEN*, *PIK3CA* and *ESR1* mutation status. End-points included proliferation, cell signalling, cell cycle and effect on ER-mediated transcription. Two patient-derived xenografts (PDX) modelling endocrine resistance were used to assess the efficacy of vistusertib, fulvestrant or the combination on tumour progression, and biomarker studies were conducted using immunohistochemistry and RNA-seq technologies.

**Results:**

Vistusertib caused a dose-dependent decrease in proliferation of all the cell lines tested and reduced abundance of mTORC1, mTORC2 and cell cycle markers, but caused an increase in abundance of EGFR, IGF1R and ERBB3 in a context-dependent manner. ER-mediated transcription showed minimal effect of vistusertib. Combined therapy of vistusertib with fulvestrant showed synergy in two ER+ PDX models of resistance to endocrine therapy and delayed tumour progression after cessation of therapy.

**Conclusions:**

These data support the notion that models of acquired endocrine resistance may have a different sensitivity to mTOR inhibitor/endocrine therapy combinations.

## Background

The largest proportion of patients diagnosed with primary breast cancer (BC) have tumours which develop in response to the female hormone oestrogen. Classically, patients with oestrogen receptor (ER)-positive BC are treated with endocrine therapy such as aromatase inhibitors (AI), which block oestrogen synthesis, or with oestrogen antagonists such as tamoxifen or fulvestrant. Despite the efficacy of these agents, resistance to endocrine therapy remains a major clinical problem (reviewed by Ma et al. [1]). In vitro and in vivo studies suggest that cross-talk between the ER and growth factor signalling pathways can circumvent the need for steroid hormone. However, direct targeting of growth factors implicated in resistance has been met with limited success, largely as a result of tumour heterogeneity (reviewed by Johnston et al. [2]).

More recently, clinical studies have focused on targeting downstream of growth factor signalling, either by direct perturbation of PI3K/mTOR or CDK4/6 within the G1/S checkpoint. De-regulation of the PI3K/AKT/mTOR pathway has been strongly implicated in resistance to endocrine therapy. Loss of the tumour suppressor *PTEN* can lead to upregulation of PI3K activity and has been associated with resistance to tamoxifen. Furthermore, upregulation of growth factor signalling via IGFR can similarly increase activity, whilst loss of *LKB1* can activate mTOR in a growth factor-independent manner. The PI3K/AKT/mTOR pathway can directly activate ER in a ligand-independent manner via phosphorylation of AF-1 at serine 167 of the ER. Furthermore, AKT has been shown to alter the ER cistrome (genome-binding pattern) effectively changing the ER transcriptional program [3]. These bi-directional interactions between hormonal and kinase signalling pathways potentiate pro-survival signals allowing BC cells to escape endocrine therapy blockade.

Based upon these observations, targeting this pathway clinically in combination with endocrine therapy has proven attractive. The BOLERO-2 study, in which patients who had progressed on a non-steroidal AI were randomised to receive the steroidal AI exemestane alone or in combination with the mTORC1 inhibitor everolimus, showed a doubling in progression-free survival in response to the combination [4], an observation supported by the phase II TAMRAD trial, which showed everolimus in combination with tamoxifen was superior to a single agent [5].

Despite the efficacy of these agents, negative feedback loops exist downstream of mTORC1 and lead to rapid tumour re-wiring resulting in increased activation of IGFR1-dependent AKT activity, which in the long term may limit their effectiveness. In the recent years, new-generation dual mTORC1/2 inhibitors have been developed, which have the potential to negate the mTORC1-associated feedback loops [6], a concept recently tested in the MANTA trial [7].

In this study, we explored the relevance of the dual mTORC1/2 inhibitor vistusertib in endocrine-resistant and endocrine-sensitive BC cell lines, as well as in patient-derived xenograft (PDX) models, and showed combination with fulvestrant had superior anti-proliferative effects compared with fulvestrant alone. Furthermore, in a fulvestrant-resistant PDX model, vistusertib re-sensitised the tumour to the anti-proliferative effect of fulvestrant.

## Methods

### Antibodies and reagents

The following primary antibodies were used in this study for immunoblotting: pRB^ser780^ (CST-3590), pRB^ser807^ (CST-8516), total-RB (CST-9309), cyclin D1 (CST-2922), cyclin D3 (CST-2936), pAKT^ser473^ (CST-9271), pAKT^Thr308^ (CST-9275), total-AKT (CST-9272), pEGFR^Tyr1068^ (CST-3777), total-EGFR (CST-2232), pERBB2^Tyr1248^ (CST-2243), total-ERBB2 (CST-4290), pERBB3^Tyr1222^ (CST-4784), pIGF1R^Tyr1135^ (CST-3918), pS6K^Ser235/236^ (CST-2211), total-S6K (CST-2217), Raptor (CST-2280), RheB (CST-13879), p4EBP1^Thr37/46^ (CST-2855), 4EBP1 (CST-9452), pSIN1^Thr86^ (CST-14716), SIN1 (CST-12860), pER^ser167^ (CST-5587), Rictor (CST-2114) and Deptor (SCT-11816) were purchased from Cell Signaling Technology. p107 (sc-318), p130 (sc-317), total-ER (sc-8002, F-10), ERBB3 (sc-415) and IGF1R (sc-713) were purchased from Santa Cruz Biotechnology. β-tubulin (T-9026) were from Sigma-Aldrich and Ki67 from Clinisciences. The following antibodies were used for immunohistochemistry: pERK1/2^Thr202/4^ (CST-4370), pAKT^ser473^ (CST-4060), pS6K^Ser235/6^ (CST-4858), pmTOR^Ser2448^ (CST-2976) and p4EBP1^Thr37/46^ (CST-2855) were purchased from Cell Signaling Technology. Ki67 was purchased from Clinisciences. Reagents were obtained from the following sources: 17-β-oestradiol (E2) and 4-hydroxytamoxifen (4-OHT) from Sigma-Aldrich, fulvestrant from Tocris, and neratinib and vistusertib from SelleckChem.

### Cell culture

Human BC cell lines MCF7, SUM44, HCC1428 and T47D were obtained from the American Type Culture Collection, USA, and Asterand. All cell lines were banked in multiple aliquots to reduce the risk of phenotypic drift and identity confirmed using short tandem repeat (STR) analysis. Cells were routinely screened for mycoplasma contamination. They were maintained in phenol red-free RPMI1640 containing 10% fetal bovine serum (FBS) and 1 nM oestradiol (E2). Long-term oestrogen-derived (LTED) equivalents modelling relapse on an AI were generated, as reported previously [8], and were maintained in phenol red-free RPMI1640 containing 10% charcoal-dextran-stripped FBS (DCC). Tamoxifen-resistant (TAMR) MCF7 cells were generated by growing wild-type MCF7 long-term in the presence of RPMI1640 containing 10% DCC + 0.01 nM E2 + 100 nM 4-OHT. Fulvestrant-resistant (ICIR) MCF7 and MCF7 LTED cell lines were generated by growing parental cells long-term in the presence of RPMI1640 containing 10% DCC + 1 nM E2 + 100 nM fulvestrant or RPMI1640 containing 10% DCC + 100 nM fulvestrant, respectively. Palbociclib-resistant (PalboR) cell lines were generated and maintained, as previously described [9, 10]. All cell lines were stripped of steroids for 48–72 h prior to the start of experiments.

### Proliferation assays

Cells were seeded into 96-well tissue culture plates and allowed to attach overnight. Monolayers were then treated with increasing concentrations of the drugs, and after 72 h, cell viability was determined using the CellTiter-Glo® Luminescent Cell Viability Assay (Promega), according to the manufacturer’s protocol. Values were expressed as relative luminescence compared to the vehicle-treated control. Non-linear regression analysis was used to fit the curves, and IC_50_ values were calculated using PRISM 7 software (GraphPad). To determine the nature of the interaction between vistusertib and fulvestrant, combination studies were performed by using Chou and Talalay’ s constant ratio combination design and quantified using CalcuSyn software (BIOSOFT, Cambridge, UK) [11]. The combination indices (CI) were obtained by using mutually non-exclusive Monte Carlo simulations. In this analysis, CI scores significantly lower than 1 were defined as synergistic, CI > 1 as antagonistic, and CI = 1 as additive.

### Immunoblotting

All cells were grown in the presence of RPMI1640 containing 10% DCC for 3 days prior to seeding. They were seeded into dishes, allowed to attach overnight and treated with the appropriate drugs the following day. After 24-h treatment, total protein was extracted and immunoblotting carried out, as previously described [8].

### Real-time quantitative PCR

mRNA from treated cells and from HBCx34 OvaR PDX models (*n* = 30 [12]) was extracted using RNeasy Mini Kit (Qiagen), quantified and reverse-transcribed with SuperScript III First-Strand Synthesis System (Invitrogen). TaqMan gene expression assays (Applied Biosystems) were used to quantify *TFF1* (Hs00907239_m1), *PGR* (Hs01556702_m1), *GREB1* (Hs00536409_m1), *PDZK1* (Hs00275727-m1) and *ESR1* (Hs01046818_m1), *EGFR* (Hs01076090_m1), *ERBB2* (Hs01001580_m1), *ERBB3* (Hs00176538_m1), *IFG1R* (Hs00609566_m1) and/or *IRS1* (Hs00178563_m1) together with *FKBP15* (Hs00391480_m1) as a housekeeping gene to normalise the data. The relative quantity was determined using ΔΔCt, according to the manufacturer’s instructions (Applied Biosystems).

### In vivo patient-derived xenografts

HBCx22 OvaR and HBCx34 OvaR PDX models resistant to endocrine therapy were established as stated previously [12], in accordance with the French Ethical Committee. Efficacy studies were carried out to determine the anti-tumour activity of vistusertib alone and combined with fulvestrant administered over 90 days. The treatment groups (10–12 mice per arm) received either vistusertib (15 mg/kg daily by oral gavage) or fulvestrant (5 mg/mouse suspended in corn oil by weekly subcutaneous injection into the flank). These concentrations are in keeping with previous studies [6] and clinical achievable doses [13] for vistusertib. For the combination group, fulvestrant was dosed 2 h before administration of vistusertib. The control groups received both vehicles. To assess whether treatment with vistusertib alone or in combination with fulvestrant could further delay tumour progression, five mice from each group were followed for an additional 40 days after drug withdrawal.

Tumour diameters were measured using calipers, and volumes were calculated as *V* = *a* × *b*^2^/2, where ‘*a*’ is the largest diameter and ‘*b*’ is the smallest. Percent change in tumour volume was calculated for each tumour as (Vf − V0/V0) × 100, where V0 is the initial volume (at the beginning of treatment) and Vf is the final volume (at the end of treatment). Tumour regression (*R*) was defined as a decrease in tumour volume of at least 50%, taking as reference the baseline tumour volume [14].

Tumour volumes were expressed relative to the initial starting volume (relative tumour volume (RTV)). Tumour growth inhibition (TGI) from the start of treatment was calculated as the ratio of the mean RTV between the control and treated groups measured at the same time. Because the variance in mean tumour volume data increases proportionally with volume (and is therefore disproportionate between groups), data were log-transformed to limit any size dependency before statistical evaluation. Statistical significance of TGI was calculated by the paired Student *t* test by comparing the individual RTVs in the treated and control groups.

### Immunohistochemistry

In order to assess biomarker changes, a pharmacodynamic study was performed for 4 days of treatment with vistusertib, fulvestrant or a combination of the two drugs in the HBCx22 OvaR PDX model. Mice were sacrificed at 4 h after the final treatment and tumours resected. Excised tumours were fixed in 10% neutral buffered formalin and paraffin-embedded, and tissue microarrays (TMA) were built from the blocks. Three xenografts from each treatment group and two tissue cores per tumour were included in the TMA. Sections from the TMA were cut and stained for the expression of biomarkers, as previously described [12]. The immunohistochemically stained TMA sections were digitally scanned at ×20 with a Hamamatsu NanoZoomer-XR whole-slide scanner (Hamamatsu Photonics K.K., Hamamatsu, Japan). The quality of the images was checked manually, and the images were analysed with Visiopharm integrator system (VIS) version 2018.9.3.5303 (Visiopharm A/S) using VIS ready to use automated image analysis algorithms (APPs).

### RNA-seq

Excised tumours from HBCx34 OvaR PDX-sacrificed mice were used for a gene expression study (*n* = 12; 3 mice by group). Libraries were created after using TruSeq Stranded mRNA Library Prep Kit (Illumina) and sequenced using the NextSeq 500 (Illumina). RNA-seq data was aligned to the human GRCh38 reference genome using STAR Aligner (star v2.6.1a) [15]; read count for each gene was calculated with HTSeq (v0.6.1) [16]. Genes were compared for differential expression between the different treatments using edgeR [17] and were considered to be statistically expressed when the absolute fold change ≥2 and false discovery rate (FDR) <5%. These significantly expressed gene lists were subject to further functional annotation using Ingenuity Pathway Analysis (IPA) to identify altered pathways due to the corresponding treatments. For individual pathways, the Benjamini–Hochberg procedure was used to the calculate FDR in order to adjust for multiple testing. RNA-seq data supporting the findings was deposited in the NCBI (http://ncbi.nlm.nih.gov/geo/) with reference PRJNA564917.

## Results

### Inhibitory effects of vistusertib on BC cell proliferation

**Fig. 1:**
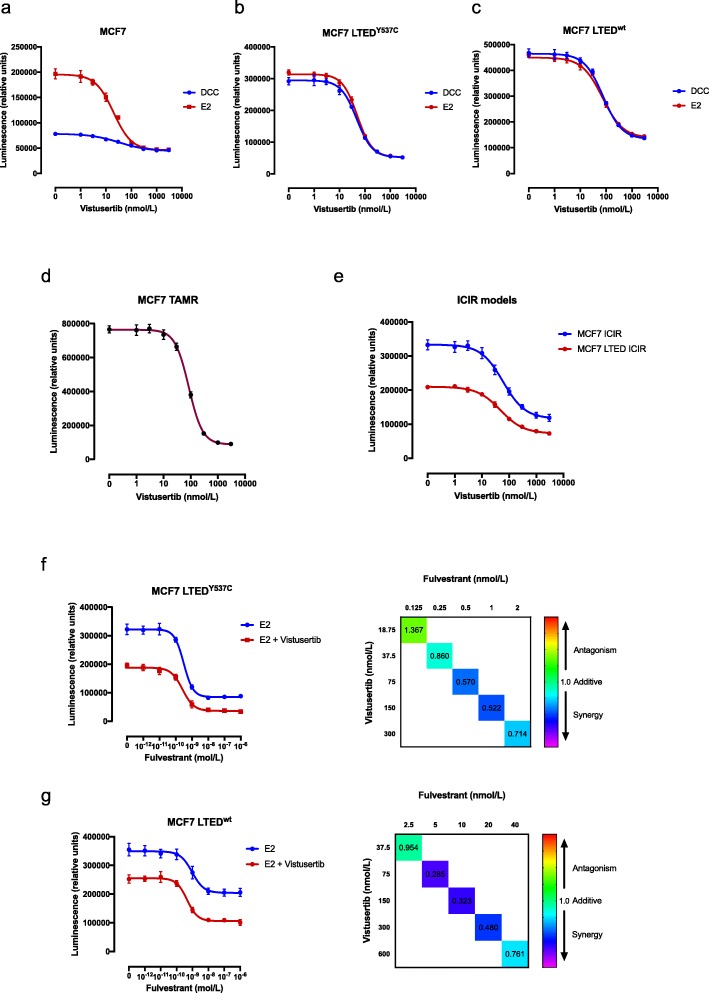
Effect of vistusertib alone or in combination with endocrine agents in several cell line models of endocrine sensitivity and resistance BC. **a**–**c** Effect of escalating doses of vistusertib on proliferation of **a** MCF7, **b** MCF7 LTED^Y537C^ and **c** MCF7 LTED^wt^ cell lines in the absence and in the presence of 0.01 nM E2. **d**, **e** Effect of escalating doses of vistusertib on proliferation of **d** tamoxifen (MCF7 TAMR)- and **e** fulvestrant (MCF7 ICIR and MCF7 LTED ICIR)-resistant cell lines. **f**, **g** Effect of escalating doses of fulvestrant in the presence or in the absence of 75 nM of vistusertib on both **f** MCF7 LTED^wt^ and **g** MCF7 LTED^Y537C^ (left panels) and respective combination index heatmaps (right panels). Data are expressed as luminescence relative to vehicle control. Cell viability was analysed using a CellTiter-Glo assay. Error bars represent mean ± SEM

We tested the anti-proliferative effect of vistusertib in a panel of isogenic cell lines modelling sensitivity or resistance to endocrine therapy (MCF7, SUM44, HCC1428 and T47D) for which the *PIK3CA*, *PTEN* and *ESR1* mutation status was previously established [18, 19]. Assays were conducted in the presence of E2, to model the effects of vistusertib as a monotherapy, or in the absence of E2, to model the combination with an AI in the primary setting. MCF7 cells showed a concentration-dependent decrease in proliferation in the presence of E2 with an IC_50_ of 20 nM. In the absence of E2, minimal further anti-proliferative effect was evident from the addition of vistusertib and the IC_50_ was increased (Fig. 1a, Additional file 1: Table S1a). In an extended panel of ER+ cell lines, in the presence of E2, vistusertib sensitivity varied with IC_50_ values between 30 and 500 nM (Additional file 2: Figure S1a and Additional file 1: Table S1a). Removal of E2 caused a drop in proliferation in all cell lines, as expected. Addition of vistusertib further reduced cell viability in a dose-dependent manner (IC_50_ values between 40 and 700 nM; Additional file 2: Figure S1a and Additional file 1: Table S1a). In order to assess the effect of vistusertib in cell lines modelling resistance to an AI, escalating concentrations were tested in two MCF7 LTED models in the presence or absence of E2. Of note, the MCF7 LTED^Y537C^, which harbour a hotspot *ESR1* mutation in the ligand-binding domain, showed sensitivity with an IC_50_ of 50 nM in the presence or absence of E2, in keeping with their ligand-independent phenotype (Fig. 1b). Contrastingly, MCF7 LTED^wt^ showed a slightly higher IC_50_ (75 nM) (Fig. 1c). Three further LTED cell lines were assessed. HCC1428 LTED expressing wild-type (wt) *ESR1*, SUM44 LTED harbouring *ESR1*^Y537S^ and T47D LTED which lose ER expression showed varying IC_50_ values between 65 and 350 nM (Additional file 2: Figure S1b and Additional file 1: Table S1a).

We further assessed sensitivity to vistusertib in cell lines modelling resistance to tamoxifen (TAMR) or fulvestrant (ICIR). In keeping with the previous data, both models showed a concentration-dependent decrease in proliferation with IC_50_ values of 85 and 50 nM, respectively (Fig. 1d, e and Additional file 1: Table S1b). Finally, we assessed the effect of escalating doses of fulvestrant in both the presence and the absence of a fixed concentration of vistusertib in MCF7 LTED^wt^ and MCF7 LTED^Y537C^ cell lines (Fig. 1f, g and Additional file 1: Table S1c). In both cell line models, the combination with vistusertib appeared synergistic with a combination index below 1.

These data suggest that vistusertib may provide benefit in combination with an AI in patients with de novo endocrine resistance and showed efficacy in models of acquired endocrine resistance irrespective of *ESR1* mutation status or ESR1 protein abundance.

### Effect of vistusertib on receptor tyrosine kinase and downstream signalling pathways

**Fig. 2:**
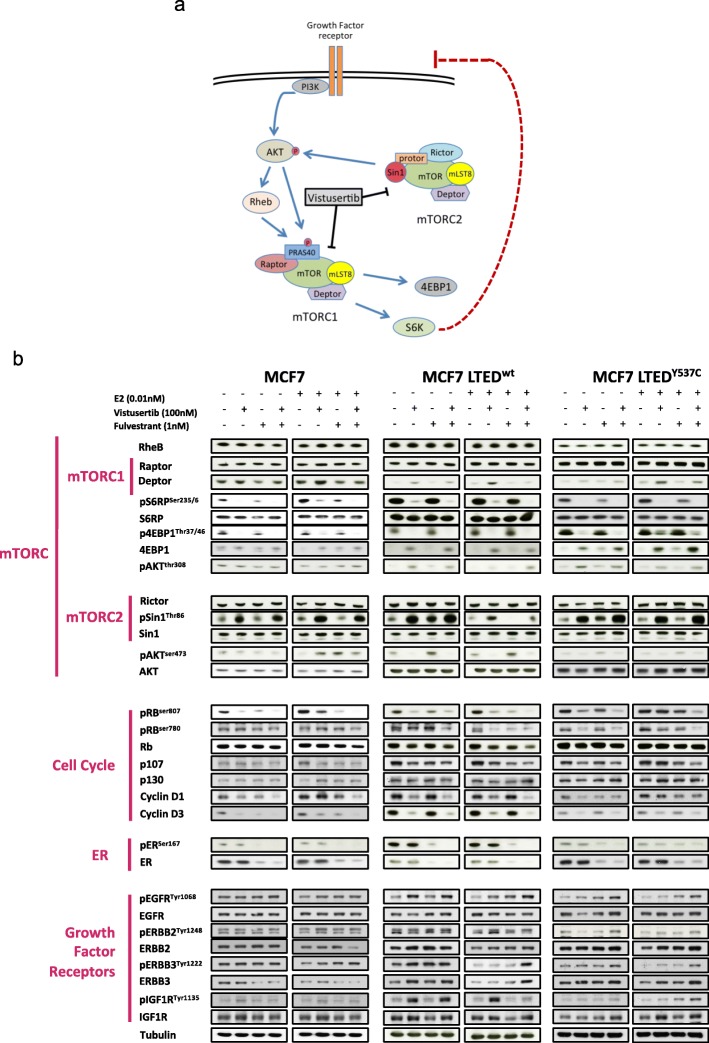
Effect of vistusertib on RTKs and downstream signalling pathways. **a** Schematic representation of the PI3K/AKT/mTOR signalling pathway and cross-talk with RTKs. **b** Effect of vistusertib alone on in combination with fulvestrant on mTORC1, mTORC2, cell cycle, ER and RTK targets, both in the presence and in the absence of 0.01 nM E2

Previous studies have shown that blockade of mTORC1 can lead to feedback loops via IGFR and ERBB signalling networks [20, 21] (Fig. 2a). In order to test the effect of targeting both mTORC1 and mTORC2, we examined the effect of vistusertib upon key protein targets within the mTOR pathway. Immunoblot analysis of the MCF7 and LTED derivatives was assessed (Fig. 2b). Vistusertib caused a decrease in the expression of pS6RP^Ser235/6^, p4EBP1^Thr37/46^ and pAKT^Ser473^ and an increase in Deptor and pSin1 together with a decrease in abundance of Cyclin D1, D3 and pRB indicative of cell cycle arrest. Treatment with fulvestrant alone or in combination with vistusertib reduced abundance of both phosphorylated and total ER. Despite the dual blockade of mTORC1/2, feedback loops via IGF1R and ERBB family members were evident but appeared cell line-specific. For instance, MCF7 LTED^wt^ showed marked increases in pIGF1R and pAKT^Thr308^ in response to vistusertib. To test if the effect of vistusertib was persistent beyond a 24-h period, we performed a time course experiment and showed a gradual increase in abundance of pEGRF, pIGF1R and pSin1 markers up to 96 h of treatment (Additional file 3: Figure S2).

### Effect of vistusertib alone or in combination with fulvestrant on ER-mediated transcription

**Fig. 3:**
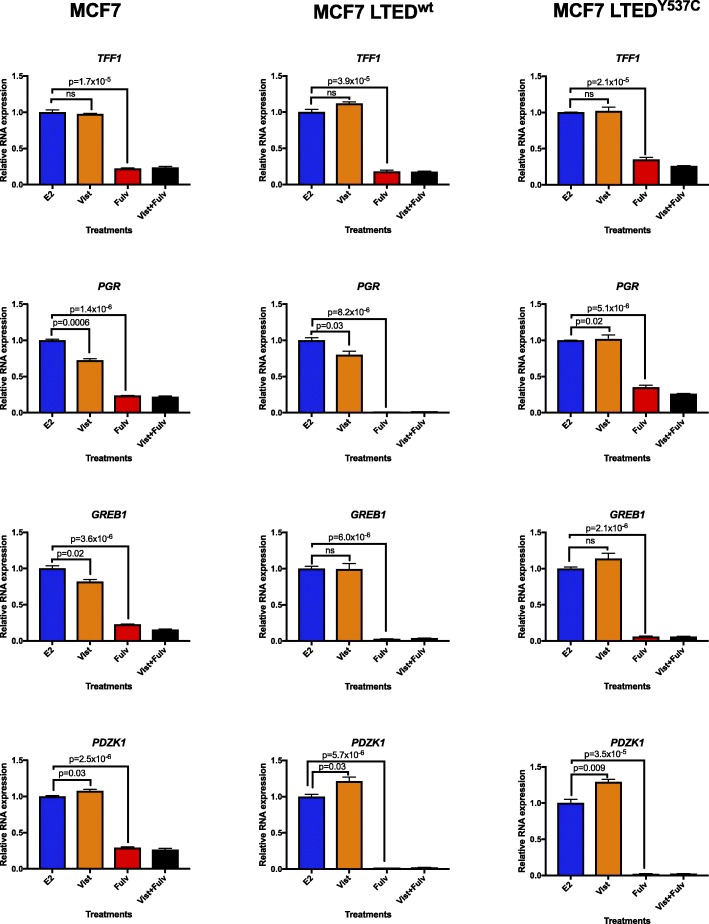
Effect of vistusertib alone or in combination with fulvestrant in ER-mediated transcription. MCF7, MCF7 LTED^wt^ and MCF7 LTED^Y537C^ were treated in the presence of 0.01 nM E2 with vistusertib, fulvestrant or the combination for 24 h, and effects on *TFF1*, *PGR*, *GREB1* and *PDZK1* were assessed by RT-qPCR. Error bars represent means ± SEM. Vist vistusertib, Fulv fulvestrant, Vist+Fulv combination treatment

Evidence suggests that cross-talk between PI3K/AKT/mTOR impacts on ER function as a transcription factor. Indeed, mTORC1 via S6RP has been shown to phosphorylate ER at serine 167 [22]. We therefore assessed the effects of vistusertib on ER-mediated transcription. The relative expression of a panel of oestrogen-regulated genes (ERGs: *TFF1*, *PGR*, *GREB1* and *PDZK1*) was evaluated in the presence or absence of E2. In MCF7 and in both MCF7 LTED derivatives, treatment with vistusertib under DCC conditions caused subtle or no changes in the expression of ERGs that was gene- and cell-specific (Additional file 4: Figure S3). Similarly, in the presence of 0.01 nM of E2, vistusertib caused small changes in the expression of the ERGs for all the three cell lines tested, but fulvestrant alone or in combination with vistusertib consistently reduced the expression of all the ERGs when compared with the vehicle control (Fig. 3). These data suggest that vistusertib does not impact ER-mediated transcription.

### Vistusertib in combination with fulvestrant impedes tumour progression in human BC PDX models of acquired endocrine resistance

**Fig. 4:**
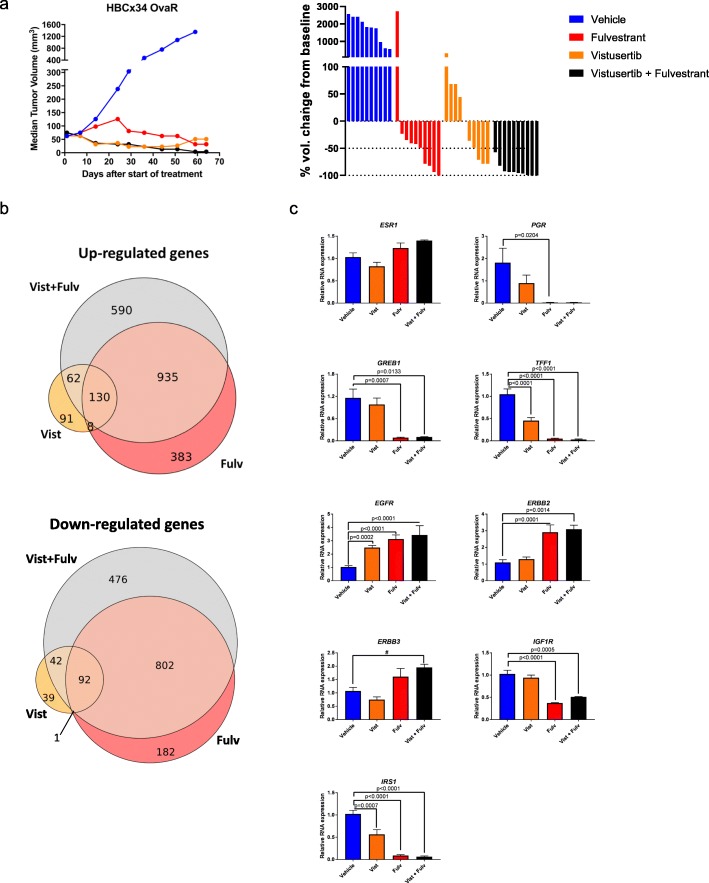
Effect of vistusertib alone or in combination with fulvestrant on tumour progression in HBCx34 OvaR PDX models. **a** Long-term study assessing changes in tumour volume over 64 days of treatment in HBCx34 OvaR. HBCx34 OvaR is an ER+ PDX model which is resistant to E-deprivation and tamoxifen but sensitive to the anti-proliferative effects of fulvestrant. Mice were treated with vehicle control, fulvestrant, vistusertib or the combination, and data shows median tumour volume (mm^3^). Bars represent the percentage of volume change at the end of treatment compared with baseline, for each individual animal. **b** Venn diagram showing the intersect of genes up- and downregulated for the different treatments by RNA-seq analysis; tumours of three animals by group were evaluated. **c** Effect of vistusertib (*n* = 10), fulvestrant (*n* = 8) or the combination (*n* = 3) in relation to vehicle (*n* = 9) upon relative RNA expression of ERGs and RTKs by RT-qPCR. Error bars represent means ± SEM. Statistical analysis was performed using ANOVA with Dunnett’s multiple comparisons test. ^#^Tendency to difference between groups by *t* test. Vist vistusertib, Fulv fulvestrant, Vist + Fulv combination treatment

In order to assess the effect of vistusertib alone or in combination with fulvestrant in vivo, we adopted two PDX models of acquired endocrine-resistant BC. HBCx34 OvaR is an ER+ PDX which is resistant to E-deprivation and tamoxifen but sensitive to the anti-proliferative effects of fulvestrant [12] (Fig. 4). After a period of 64 days, all treatments showed over a 95% reduction in tumour volume (fulvestrant: 97.6%, *p* = 0.004; vistusertib: 96.2%, *p* < 0.0001; combination: 99.7%, *p* < 0.0001) compared to vehicle control (Fig. 4a and Additional file 5: Figure S4). Vistusertib showed greater efficacy than fulvestrant as a monotherapy over the first 50 days (adjusted *p* value = 0.005) and appeared similar to the combination over this time period. At the end of treatments, all xenografts were in regression or complete response in the combination arm (percentage of tumour volume change ≤50%), against four xenografts in the fulvestrant-treated group (Fig. 4a).

Analysis of the combination of vistusertib and fulvestrant appeared the most effective, showing a significant increase in efficacy compared to fulvestrant alone (*p* = 0.0001, Mann–Whitney test, Fig. 4a).

In order to further explore the impact of vistusertib alone or in combination with fulvestrant, tumours were resected at the end of the study and subjected to RNA-seq. Fulvestrant showed the greatest impact on gene expression (1456 upregulated and 1077 downregulated genes) versus vistusertib (291 upregulated and 174 downregulated genes) when compared with vehicle control (Fig. 4b). Noteworthy, the number of gene changes as a result of the combination largely reflected that seen for fulvestrant (1717 upregulated and 1412 downregulated genes) indicating the mitogenic driver within this PDX remains ER. In order to identify canonical pathways affected by these treatments, we conducted ingenuity pathway analysis (IPA; FDR <5%) using differentially expressed genes (FDR <5% and fold change ≥2; Additional file 6: File S1). Fulvestrant showed a dominant effect on cell cycle and oestrogen-mediated S-phase entry both as a monotherapy or in combination with vistusertib. Contrastingly, single-agent vistusertib showed no impact on ER-mediated S-phase entry. Treatment with vistusertib showed minimal although significant enrichment of EGF, ERBB and ERK/MAPK signalling compared with vehicle control (Additional file 6: File S1). In order to explore this further, we carried out targeted qRT-PCR (Fig. 4c). Treatment with fulvestrant significantly reduced the expression of *TFF1*, *PGR*, *GREB1* and *IRS1* but increased the expression of *EGFR*, *ERBB2* and *ERBB3*. Contrastingly, vistusertib had minimal effect on the expression of *ESR1*, *GREB1* and *PGR*; however, it significantly reduced *TFF1* but not to the degree seen with fulvestrant or the combination. Noteworthy, vistusertib significantly increased the expression of *EGFR* but not *ERBB2*, *ERBB3* or *IGF1R*.

**Fig. 5:**
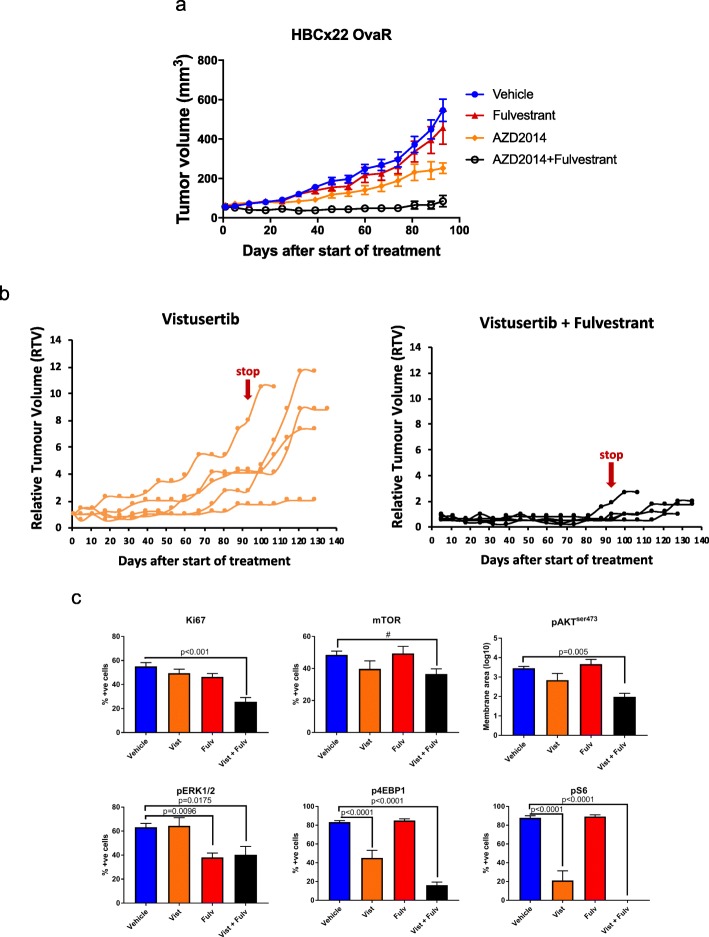
Effect of vistusertib alone or in combination with fulvestrant on tumour progression in HBCx22 OvaR PDX models. **a** Long-term study assessing changes in tumour volume over 93 days of treatment in HBCx22OvaR. HBCx22 OvaR is an ER+ model that shows partial resistance to fulvestrant. Mice were treated with vehicle control, fulvestrant, vistusertib or the combination. Data represents mean relative tumour volume ± SEM. **b** Effect of vistusertib alone or in combination with fulvestrant on tumour growth of individual mice over a period of 93 days. Treatments were withdrawn and tumour growth reassessed for a further 40 days to establish the efficacy of the drugs in delaying tumour progression. **c** Immunohistochemical analysis of several markers following treatment for a period of 4 days with either vehicle, vistusertib (Vist), fulvestrant (Fulv) or the combination of both (Vist + Fulv). Tumours were harvested 4 h after the last treatment. Statistical analysis was performed using ANOVA with Dunnett’s multiple comparisons test. ^#^Tendency to difference between groups by *t* test

In order to further explore the efficacy of the combination of vistusertib with fulvestrant, a second PDX model, HBCx22 OvaR, was assessed. HBCx22 OvaR is an ER+ model showing partial resistance to fulvestrant and harbours a 24-base-pair in-frame deletion in exome 13 in *PIK3R1* [12] (Fig. 5). As expected, single-agent fulvestrant had no significant impact on tumour progression compared to vehicle control, confirming the resistant phenotype. Vistusertib as a monotherapy delayed tumour progression by 54.5% (*p* = 0.04) compared to vehicle control. The combination of vistusertib plus fulvestrant was the most effective treatment with tumour volumes 84.7% lower than vehicle control (*p* = 0.0002) (Fig. 5a). After 93 days of treatment, the therapies were withdrawn and the tumour volumes assessed for a further 40 days in order to establish the efficacy of the drugs in delaying tumour progression (Fig. 5b). Removal of therapies showed sustained anti-tumour effect in the combination group, whilst tumours treated with vistusertib alone showed significant progression.

In order to assess dynamic changes, three mice per arm were sacrificed after 4 days of therapy and tissue sections were subjected to immunohistochemical analysis. Treatment with vistusertib or vistusertib in combination with fulvestrant revealed suppression of pAKT^Ser473^, p4EBP1^Thr37/46^ and pS6RP^Ser235/6^, as well as a slight but noticeable decrease in pmTOR^Ser2448^ (Fig. 5c and Additional file 7: Figure S5a). Furthermore, fulvestrant reduced the expression of pERK1/2^Thr202/4^ both alone and in combination with vistusertib. In contrast to our in vitro analysis, no alteration in the abundance of pEGFR and pIGFR was evident in response to vistusertib alone, whilst pEGFR was significantly suppressed by the combination with fulvestrant (Additional file 7: Figure S5b). Noteworthy, assessment of Ki67 showed the greatest reduction when the combination of vistusertib and fulvestrant was used (Additional file 7: Figure S5a).

Taken together, these data suggest the combination may provide greater efficacy than fulvestrant alone in ER+ acquired endocrine-resistant disease.

### Effectiveness of vistusertib in combination with pan-ERBB inhibitors and in models of resistance to palbociclib

**Fig. 6:**
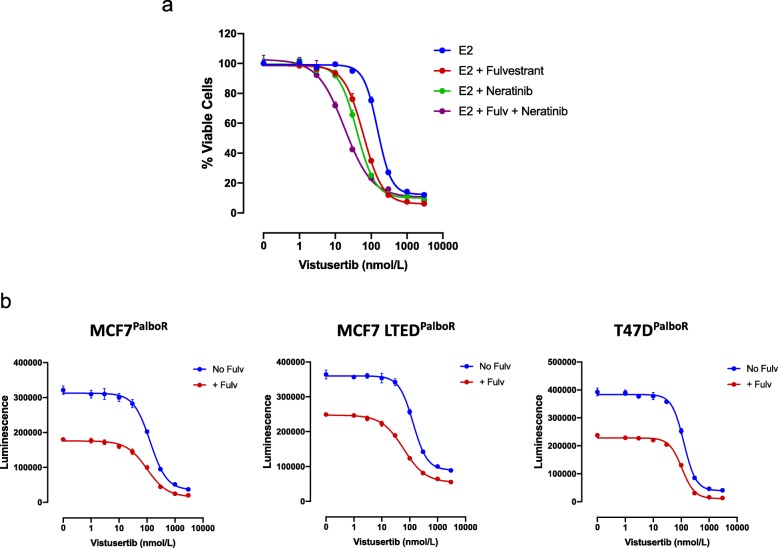
Effect of vistusertib in combination with neratinib/fulvestrant in cell line models of endocrine- and palbociclib-resistant BC. **a** Effect of escalating doses of vistusertib in combination with fulvestrant (1 nM) (Fulv) and neratinib (500 nM) on proliferation of MCF7 LTED^wt^ cell lines in the presence of 0.01 nM E2. Data are expressed as the percentage of viable cells relative to vehicle control. **b** Effect of escalating doses of vistusertib with or without fulvestrant (1 nM) on proliferation of palbociclib-resistant MCF7^PalboR^, MCF7 LTED^PalboR^ and T47D^PalboR^ cell lines. Data are expressed as luminescence. Error bars represent mean ± SEM

As increased feedback loops via ERBB and IGF1R family members were evident in vitro and from our gene expression analysis, we assessed sensitivity of MCF7-LTED^wt^ cell lines to the anti-proliferative effect of vistusertib, or fulvestrant combined with the pan-ERBB inhibitor neratinib, or the combination of all three agents (Fig. 6a). Fulvestrant and neratinib enhanced the anti-proliferative effect of vistusertib; however, the triple combination was the most effective. These data further support previous observations in which the triple combination targeting three cellular nodes: ERBB, ER and mTORC1, showed the greatest anti-proliferative effect [20].

More recently, CDK4/6 inhibitors have become the standard of care in the treatment of endocrine-resistant ER+ BC. Despite their efficacy, not all patients benefit and many will eventually relapse with acquired resistance. Studies suggest that cross-talk exists between CDK4 and the mTOR pathway via pTSC2 [23] and that blockade of mTORC1/2 may delay the onset of resistance to CDK4/6 inhibition [24]. To assess this, we treated three palbociclib-resistant cell line models (MCF7-^PalboR^, MCF7 LTED-^PalboR^ and T47D-^PalboR^) (Fig. 6b) with escalating concentrations of vistusertib with or without fulvestrant. All three cell lines showed sensitivity to mTORC1/2 blockade. The addition of fulvestrant further enhanced the anti-proliferative effect. Taken together, these data suggest mTORC1/2 blockade remains effective after acquisition of resistance to palbociclib.

## Discussion

Cross-talk between the PI3K/AKT/mTOR pathway and ER is well documented, and targeting this pathway with the mTORC1 inhibitor everolimus has shown marked efficacy [25]. However, negative feedback loops have been identified leading to activation of growth factor signalling pathways and reduced drug sensitivity [20, 21]. In this study, we assessed the efficacy of the mTORC1/2 inhibitor vistusertib in vitro and in PDX models of endocrine resistance. In summary, we show that vistusertib as a monotherapy had little impact on global gene expression compared to fulvestrant and did not significantly impact ER-mediated transactivation. These findings are in contrast to previous studies which have shown that inhibition of PI3K leads to an open chromatin state at oestrogen target loci resulting in enhanced ER-mediated transactivation, supporting the concept of combined PI3K and endocrine therapies [26]. However, our observations are in keeping with a recent study which explored the impact of mTORC suppression on the genome-wide recruitment of ER, which showed no alteration in binding patterns compared to vehicle control [24]. This would suggest that direct cross-talk may be restricted to PI3K and AKT [3, 26].

Vistusertib as a single agent significantly suppressed the abundance of pS6 and p4EBP1 both in vitro and in vivo. In contrast to our previous studies with everolimus [20, 21], vistusertib decreased the abundance of pAKT^ser473^, whilst increasing pAKT^thr308^ indicative of efficient suppression of both mTORC1 and mTORC2 activity. In addition, AZD2014 may display different target engagement properties from everolimus, which may in turn lead to different clinical efficacy. Nonetheless, we found evidence of increased expression of pEGFR and pIGF1R in a context-specific manner, suggesting that tumour re-wiring and feedback loops previously associated with poor response to mTORC1 suppression were evident. However, despite this, cell proliferation was significantly reduced both in vitro and in vivo. Moreover, the enhanced expression of growth factor receptors, in particular members of the ERBB family, was far more pronounced with fulvestrant.

There are two underlying mechanisms by which EGFR can be increased in this context. Firstly, suppression of mTOR leads to loss of phosphorylated TCS2 and suppression of S6, leading to the removal of the negative feedback loop resulting in increased expression of EGFR [23]. Conversely, ER is known to cross-talk with EGFR/ERBB2, and studies suggest that ER sequesters the coactivators AIB1 and SRC1, leading to the suppression of ERBB2 signalling, whilst in the presence of fulvestrant, downregulation of ER function would lead to the converse [27, 28]. Despite this early re-wiring, the combination of vistusertib and fulvestrant showed enhanced anti-tumour activity which was maintained even after cessation of the drug in the PDX model resistant to fulvestrant.

It is noteworthy, in our HBCx34 model which is PTEN competent and ER+, that ER expression remains the dominant mitogenic driver. In this context, mTORC1/2 suppression is sufficient to impede tumour progression, most likely as the PI3K pathway is not hyperactivated. In addition, this PDX is sensitive to fulvestrant, and thus, combining blockade of ER and mTORC1/2 significantly impedes tumour progression. Contrastingly, HBCx22 shows hyperactivation of the PI3K/AKT/mTOR pathway as a result of a *PIK3R1* frameshift and, despite continuing to express high levels of ER, is resistant to fulvestrant. In this setting, monotherapy targeting ER or mTORC1/2 is insufficient to have prolonged anti-tumour effect whilst the combination targeting both pathways suppresses tumour progression even after cessation of therapy.

The recent MANTA trial explored the concept of targeting both ER and mTORC1/2 in patients with primary and secondary AI therapy-resistant disease. The patients were randomised to single-agent fulvestrant versus fulvestrant in combination with vistusertib or everolimus. Although not significant, the combination of vistusertib plus fulvestrant showed a trend towards improved progression-free survival in the first year compared to fulvestrant as a single agent (median 7.6–8.0 versus 5.4 months). However, the combination of fulvestrant plus everolimus appeared superior, increasing progression-free survival from 5.4 to 12.3 months [7]. The lack of a significant effect of the combination of vistusertib plus fulvestrant compared to everolimus may reflect the differences in target engagement properties for the two compounds, or alternatively different dependency of patients who have relapse on AI therapy on mTORC1 signalling. These data are in contrast to those seen in our PDX models, and one explanation could be that prior treatment influences responses to secondary combinations. For instance, the most powerful anti-proliferative effects seen in our study were associated with resistance to fulvestrant. This suggests that in patients with acquired resistance, previous lines of endocrine therapy should be considered to guide treatment choices.

Lastly, as noted, CDK4/6 inhibitors are changing the face of therapy for ER+ BC ([29, 30]; however, not all patients will respond and many will acquire resistance. Previous studies have shown that the combination of mTORC1/2 inhibition with a CDK4/6 inhibitor enhances E2F suppression and delays the onset of resistance as well as circumventing it [24]. In order to corroborate these observations, we assessed vistusertib sensitivity in a panel of cell lines with acquired resistance to palbociclib [9, 10]. Unlike the previous study, our cell lines utilised different resistance mechanisms including loss of RB copy number (T47D-^PalboR^) and tumour re-wiring via increased growth factor signalling (MCF7-^PalboR^ and MCF7-LTED^PalboR^). Vistusertib effectively suppressed the proliferation of all models tested, and this effect was enhanced by the addition of fulvestrant. These data provide further support for the concept that mTORC1/2 inhibitors may provide utility after acquisition of resistance to CDK4/6 inhibitors.

## Conclusions

In summary, our data suggests that suppression of mTORC1 and mTORC2 has no significant impact on ER-mediated transcription, but combination therapy with fulvestrant shows synergistic benefit. Patients with secondary acquired resistant ER+ BC may have different sensitivities to mTOR inhibition in combination with endocrine therapy. Finally, mTORC1/2 inhibitors may provide utility after relapse on CDK4/6 inhibitors.

## Supplementary information

**Additional file 1: Table S1a-c.** IC_50_ values for antiproliferative effect of (**a**) vistusertib for several endocrine sensitive and resistant cell line models both in the presence or absence of 0.01 nM E2, (**b**) vistusertib in cell line models of resistance to tamoxifen (TAMR) and fulvestrant (ICIR); (**c**) fulvestrant alone or in combination with 75 nM of vistusertib in the presence of 0.01 nM E2.Table S1b. IC_50_ values for antiproliferative effect of vistusertib in cell line models models of resistance to tamoxifen (TAMR) and fulvestrant (ICIR).Table S1c. IC_50_ values for antiproliferative effect of fulvestrant alone or in combination with 75 nM of vistusertib in the presence of 0.01 nM E2.

**Additional file 2: Figure S1**. Effect of vistusertib in models of endocrine sensitive and resistant BC. **(a)** Effect of escalating doses of vistusertib on proliferation of endocrine sensitive (HCC1428, T47D and SUM44) and (**b**) endocrine resistant (HCC1428 LTED, T47D LTED and SUM44 LTED^Y537S^) cell line models both in the absence and in the presence of 0.01 nM E2. Data are expressed as relative luminescence and represented as fold-change relative to vehicle DCC control for each cell line condition.

**Additional file 3: Figure S2.** Effect of vistusertib on RTKs and downstream signalling pathways over a time course of 96 hours. MCF7 LTED^wt^ were treated for a time-course period of 24, 48, 72 and 96 hours with or without vistusertib (100 nM) in the presence or absence of E2 (0.01 nM).

**Additional file 4: Figure S3.** Effect of vistusertib in ER-mediated transcription. MCF7, MCF7 LTED^wt^ and MCF7 LTED^Y537C^ were treated in the absence of E2 with vehicle or vistusertib for 24 hours and effects on *TFF1, PGR, GREB1* and *PDZK1* were assessed by RT-qPCR (*n* = 2 biological and *n* = 3 technical replicates). Error bars represent means ± SEM. Note, as MCF7 LTED^wt^ do not express *PGR*, this was excluded from the analysis.

**Additional file 5: Figure S4.** Effect of vistusertib alone or in combination with fulvestrant on tumour progression in HBCx34 OvaR PDX models**. (a)** Assessment of tumour volume in individual animals treated with vehicle, fulvestrant, vistusertib or the combination.

**Additional file 6: File S1.** Ingenuity pathway analysis of the HBCx34 OvaR PDX models at the end of the study.

**Additional file 7: Figure S5.** Representative immunohistochemistry images of (a) expression of Ki67, mTOR, pAKT^ser473^, p4EBP1, pS6 and pERK1/2 and (b) pEGFR and pIGF1R in HBCx22 OvaR PDX models following treatment for a period of 4 days with either vehicle, vistusertib (Vist), fulvestrant (Fulv) or the combination of both (Vist + Fulv).

## Data Availability

RNA-seq data supporting the finding from this manuscript was deposited in the NCBI (http://ncbi.nlm.nih.gov/geo/) with reference PRJNA564917.

## Abbreviations

ER: Oestrogen receptor
ER+: Oestrogen receptor positive
BC: Breast cancer
AI: Aromatase inhibitors
E2: Oestradiol
LTED: Long-term oestrogen deprived
FBS: Fetal bovine serum
DCC: Dextran charcoal
4-OHT: 4-Hydroxytamoxifen
RTK: Receptor tyrosine kinase
PDX: Patient-derived xenografts
RTV: Relative tumour volume
TGI: Tumour growth inhibition
TMA: Tissue microarray
TAMR: Tamoxifen resistance
ICIR: Fulvestrant resistance
ERGs: Oestrogen-regulated genes
