# Correction to: Combination of mTORC1/2 inhibitor vistusertib plus fulvestrant in vitro and in vivo targets oestrogen receptor-positive endocrine-resistant breast cancer

**DOI:** 10.1186/s13058-020-1254-5

**Published:** 2020-01-31

**Authors:** Sunil Pancholi, Mariana Ferreira Leal, Ricardo Ribas, Nikiana Simigdala, Eugene Schuster, Sophie Chateau-Joubert, Lila Zabaglo, Margaret Hills, Andrew Dodson, Qiong Gao, Stephen R. Johnston, Mitch Dowsett, Sabina C. Cosulich, Elisabetta Marangoni, Lesley-Ann Martin

**Affiliations:** 1 Breast Cancer Now Toby Robins Research Centre, The Institute of Cancer Research, London, SW7 3RP UK; 2 BioPole Alfort, Ecole Nationale Veterinaire d’Alford, Maisons Alfort, France; 3 Ralph Lauren Centre for Breast Cancer Research, Royal Marsden Hospital, London, SW3 6JJ UK; 4 Breast Unit, Royal Marsden Hospital, London, SW3 6JJ UK; 5 Bioscience, Oncology, IMED Biotech Unit, AstraZeneca, Cambridge, UK; 6 Department of Translational Research, Institut Curie, Paris, France

**Correction to: Breast Cancer Research (2019) 21:135**

**https://doi.org/10.1186/s13058-019-1222-0**

After publication of the original article [1], we were notified that an author’s surname has been erroneously spelled. Elisabetta Maragoni’s family name should be replaced with Marangoni.
