# Supplementary material for: Combination of mTORC1/2 inhibitor vistusertib plus fulvestrant in vitro and in vivo targets oestrogen receptor-positive endocrine-resistant breast cancer
Source: Breast Cancer Res. 2019 Dec 4;21:135. doi: 10.1186/s13058-019-1222-0 (PMC6894349; doi:10.1186/s13058-019-1222-0)
Supplement: Supplementary file 1 — Additional file 1: Table S1a-c. IC50 values for antiproliferative effect of (a) vistusertib for several endocrine sensitive and resistant cell line models both in the presence or absence of 0.01 nM E2, (b) vistusertib in cell line models of resistance to tamoxifen (TAMR) and fulvestrant (ICIR); (c) fulvestrant alone or in combination with 75 nM of vistusertib in the presence of 0.01 nM E2.Table S1b. IC50 values for antiproliferative effect of vistusertib in cell line models models of resistance to tamoxifen (TAMR) and fulvestrant (ICIR).Table S1c. IC50 values for antiproliferative effect of fulvestrant alone or in combination with 75 nM of vistusertib in the presence of 0.01 nM E2. [file 13058_2019_1222_MOESM1_ESM.docx]

**Table 1a. IC_50_ values for antiproliferative effect of vistusertib for several endocrine sensitive and resistant cell line models both in presence or absence of 0.01nM E2.**

| **IC_50_ values (nmol/L)** | **DCC** | **E2 (0.01nM)** |
| --- | --- | --- |
| **MCF7** | 33.9 | 19.8 |
| **MCF7 LTED^wt^** | 76.6 | 72.6 |
| **MCF7 LTED^Y537C^** | 47.1 | 50.1 |
| **HCC1428** | 55.2 | 73.9 |
| **HCC1428 LTED** | 120.2 | 120.6 |
| **T47D** | 53.3 | 26.8 |
| **T47D LTED** | 67.8 | 62.3 |
| **SUM44** | 676 | 497 |
| **SUM44 LTED^Y537S^** | 356.2 | 169.4 |

**Table 1b. IC_50_ values for antiproliferative effect of vistusertib in cell line models models of resistance to tamoxifen (TAMR) and fulvestrant (ICIR).**

| **IC_50_ values (nmol/L)** |  |
| --- | --- |
| **MCF7 TAMR** | 84.5 |
| **MCF7 ICIR** | 59.7 |
| **MCF7 LTED ICIR** | 47.9 |

**Table 1c. IC_50_ values for antiproliferative effect of fulvestrant alone or in combination with 75nM of vistusertib in the presence of 0.01nM E2.**

| **IC_50_ values (mol/L)** | **E2** | **E2 plus vistusertib** |
| --- | --- | --- |
| **MCF7 LTED^wt^** | 9.4 x 10^-10^ | 4.5 x 10^-10^ |
| **MCF7 LTED^Y537C^** | 3.1 x 10^-10^ | 2.6 x 10^-10^ |
