# Supplementary figures and images for: Combination of mTORC1/2 inhibitor vistusertib plus fulvestrant in vitro and in vivo targets oestrogen receptor-positive endocrine-resistant breast cancer
Source: Breast Cancer Res. 2019 Dec 4;21:135. doi: 10.1186/s13058-019-1222-0 (PMC6894349; doi:10.1186/s13058-019-1222-0)

a

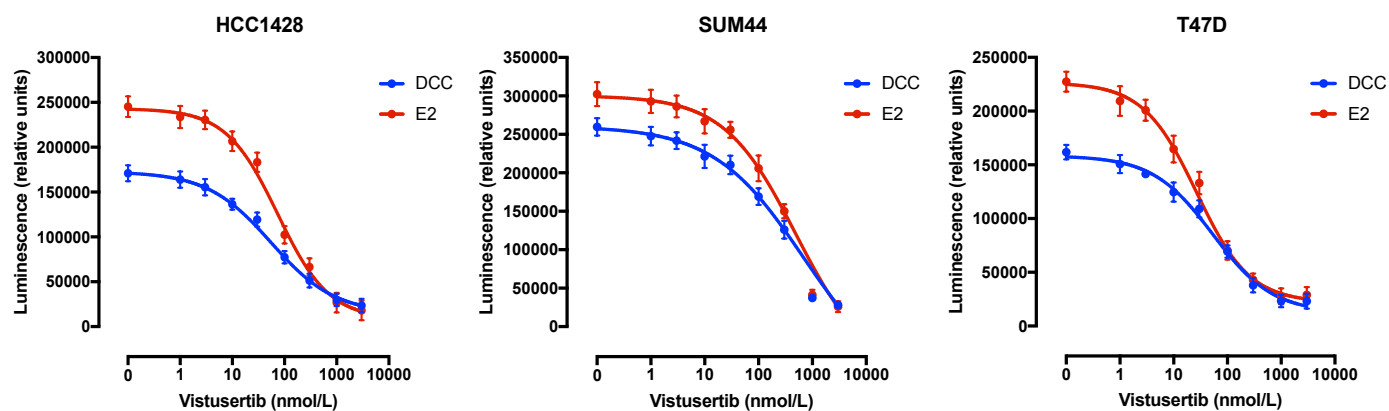

b

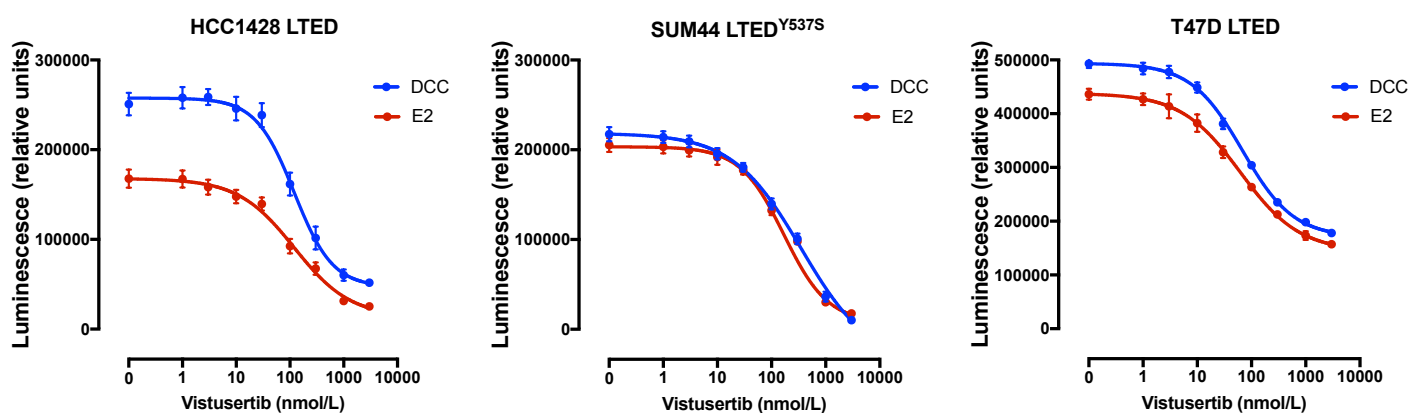

Figure S1

Supplement: Supplementary file 2 — Additional file 2: Figure S1. Effect of vistusertib in models of endocrine sensitive and resistant BC. (a) Effect of escalating doses of vistusertib on proliferation of endocrine sensitive (HCC1428, T47D and SUM44) and (b) endocrine resistant (HCC1428 LTED, T47D LTED and SUM44 LTEDY537S) cell line models both in the absence and in the presence of 0.01 nM E2. Data are expressed as relative luminescence and represented as fold-change relative to vehicle DCC control for each cell line condition. [file 13058_2019_1222_MOESM2_ESM.pdf]

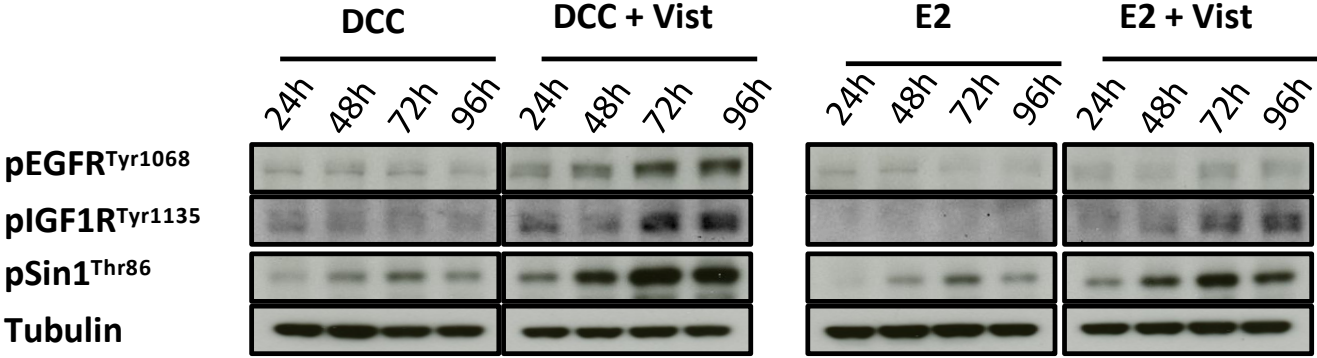

Figure S2

Supplement: Supplementary file 3 — Additional file 3: Figure S2. Effect of vistusertib on RTKs and downstream signalling pathways over a time course of 96 hours. MCF7 LTEDwt were treated for a time-course period of 24, 48, 72 and 96 hours with or without vistusertib (100 nM) in the presence or absence of E2 (0.01 nM). [file 13058_2019_1222_MOESM3_ESM.pdf]

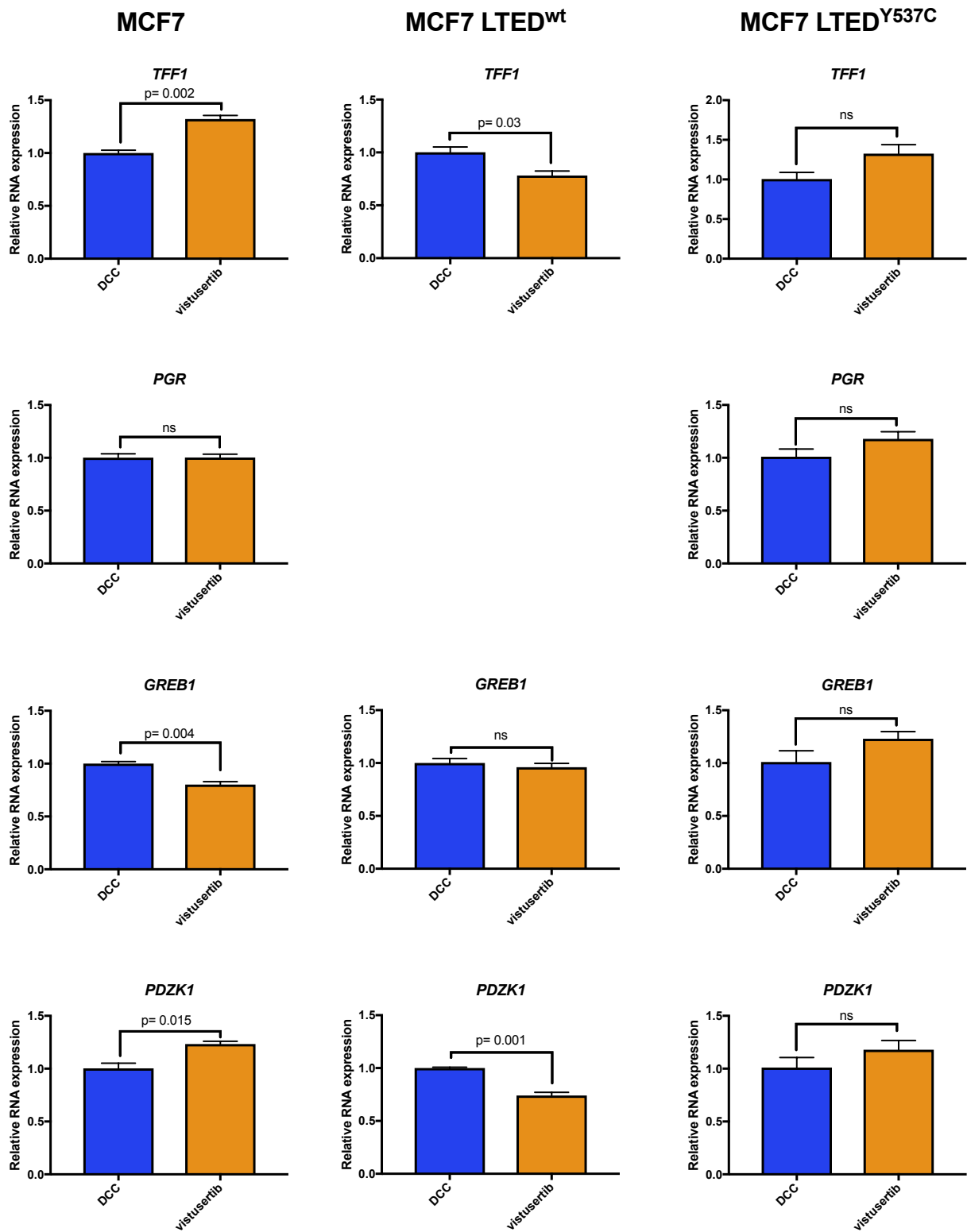

Figure S3

Supplement: Supplementary file 4 — Additional file 4: Figure S3. Effect of vistusertib in ER-mediated transcription. MCF7, MCF7 LTEDwt and MCF7 LTEDY537C were treated in the absence of E2 with vehicle or vistusertib for 24 hours and effects on TFF1, PGR, GREB1 and PDZK1 were assessed by RT-qPCR (n = 2 biological and n = 3 technical replicates). Error bars represent means ± SEM. Note, as MCF7 LTEDwt do not express PGR, this was excluded from the analysis. [file 13058_2019_1222_MOESM4_ESM.pdf]

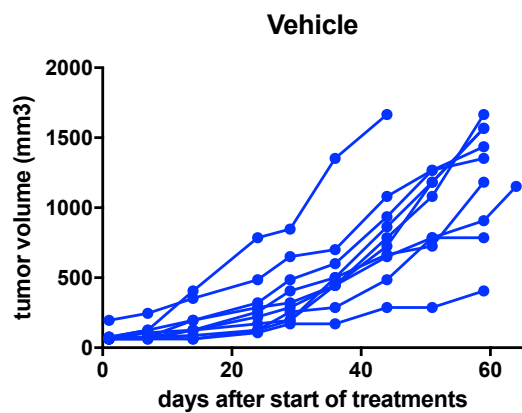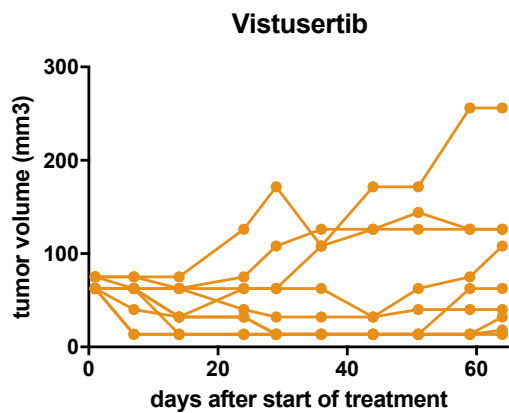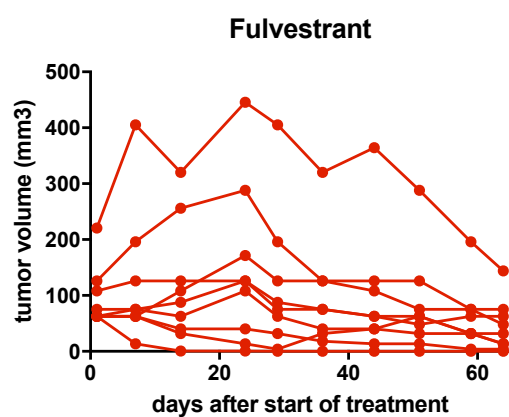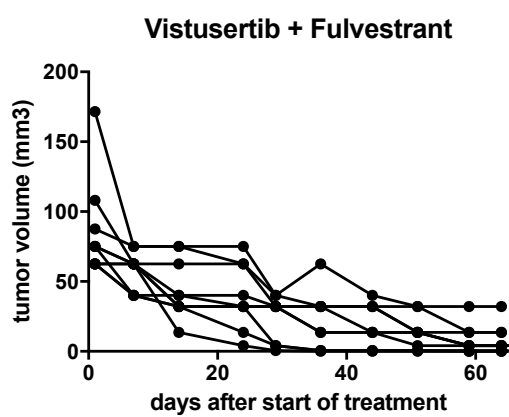

Figure S4

Supplement: Supplementary file 5 — Additional file 5: Figure S4. Effect of vistusertib alone or in combination with fulvestrant on tumour progression in HBCx34 OvaR PDX models. (a) Assessment of tumour volume in individual animals treated with vehicle, fulvestrant, vistusertib or the combination. [file 13058_2019_1222_MOESM5_ESM.pdf]

a

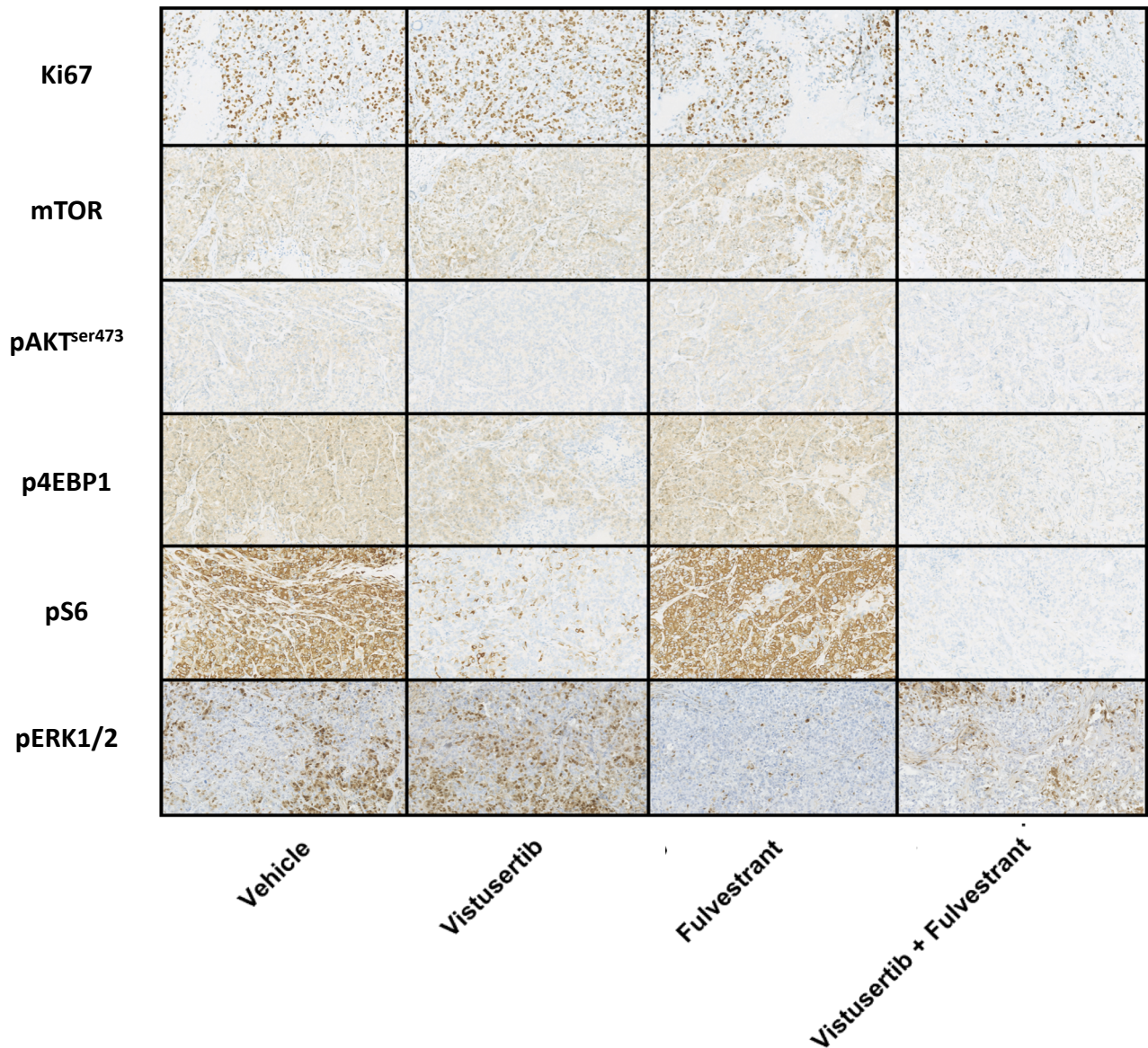

b

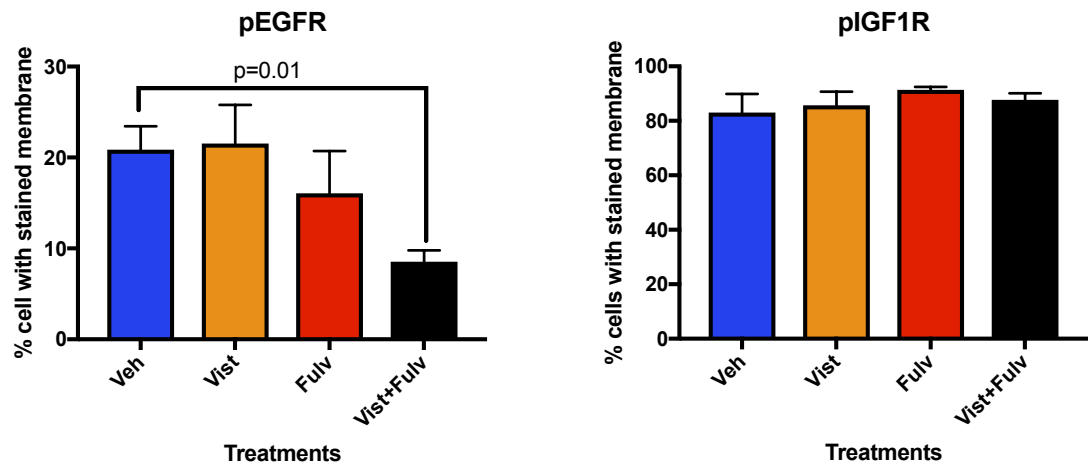

Figure S5

Supplement: Supplementary file 7 — Additional file 7: Figure S5. Representative immunohistochemistry images of (a) expression of Ki67, mTOR, pAKTser473, p4EBP1, pS6 and pERK1/2 and (b) pEGFR and pIGF1R in HBCx22 OvaR PDX models following treatment for a period of 4 days with either vehicle, vistusertib (Vist), fulvestrant (Fulv) or the combination of both (Vist + Fulv). [file 13058_2019_1222_MOESM7_ESM.pdf]
